# Supplementary material for: Understanding Primary Care Physician Vaccination Behaviour: A Systematic Review
Source: Int J Environ Res Public Health. 2022 Oct 25;19(21):13872. doi: 10.3390/ijerph192113872 (PMC9654811; doi:10.3390/ijerph192113872)
Supplement: Supplementary file 1 [file ijerph-19-13872-s001.zip › Supplementary Material S1.pdf]

Table S1: *Intrinsic and extrinsic factors reported by each study and their influence on vaccination/recommendation.*

[OV]*: Own vaccination;* ***[PR]****: patient recommendation;* ***INF*** *(influenza);* ***HPV*** *(human papillomavirus vaccine);* ***HBV*** *(hepatitis B);* ***MEAS*** *(measles);* ***MMR*** *(measles, mumps and rubella);* ***PER*** *(pertussis);* ***PNV*** *(pneumococcal vaccine);* ***PPSV23*** *(pneumococcal polysaccharide vaccine);* ***ZV*** *(zoster vaccine);* ***RVZ*** *(recombinant zoster vaccine);* ***ZVL*** *(zoster live-attenuated vaccine);* ***TT*** *(tetanus vaccine);* ***Td*** *(tetanus-diphtheria);* ***Tdap*** *(tetanus, diphtheria, pertussis).*

Author	Data and data-sources	Knowledge	Safety	Efficacy/benefits	Perceived risk	Trust	Protection	Important
Statistical hypothesis testing between KBAB and own vaccination and patient recommendation								
**Verger P et al. [21]**	-- -- --	-- -- --	Safe **OR: 1.93[1.54-2.31]**	-- -- --	No perceived risk **OR:0.47[0.20-0.74]**	Trust in institutions **OR:0.86**	-- -- --	-- -- --
**Verger P et al. [22]**	-- -- --	-- -- --	Safe **OR:0.27 [0.09-0.81]**	-- -- --	Perceived Risk **OR: 7.56[2-20-26.03]**	Trust in institutions **OR: 0.10[0.04-0.23]**	-- -- --	-- -- --
**Arlt J et al. [23]**	Co-workers **OR: 2.26 [1.90-4.29]**<br>Media News **OR: 0.16[0.05-0.53]**<br>Scientific Journals (81%), Continuing Education Courses (81%), Pharmaceutical Representatives (46%), I (38%), Co-workers (21%), Media News (5%)<br>Insufficient Information (14%-21%)	Good Knowledge: 80% [**INF** (65%)]	Adverse drug reaction (85%)	-- -- --	Dangerous Disase (79%)	-- -- --	Self- Protection (55%), Patient Protection (6%) Protection family and friend(5%) [OV]	-- -- --

Author	Data and data-sources	Knowledge	Safety	Efficacy/benefits	Perceived risk	Trust	Protection	Important
**Neufeind J et al. [24]**	Official Sources **OR: 6.95[2.97-15.84] [INF]** [PR]<br>Official Sources (90%), Guides (4%), Personal Experiences (3%)	-- -- --	Safe **OR: 1.64[1.09-2.40] [INF]** [PR]<br>Safe **OR: 1.28[1-1.63] [MEAS]** [PR]<br>Safe **OR: 1.42[1.16-1.73] [INF, PER, HBV]** [OV]	No Efficacy (41%) **[INF]**<br>No Benefit-Risk (36%) **[INF]**<br>[PR]	No Perceived Risk	Trust in institutions **[OR:1.43[1.22-1.70] [INF]** [PR]<br><br>Trust in institutions (84%)	-- -- --	No Importance **OR: 0.71[0.51-0.96] [INF, PER, HBV]** [OV]
**Verhees RAF et al. [25]**		-- -- --		Efficacy **p=0.00** [OV]<br>No Efficacy	No Perceived Risk (51%) [OV]		Self -Protection (61%), Patient Protection (88%) [OV]<br>Previous protection (25%)	
**Vezzosi L et al. [26]**	Lack of information (45% [**PNV**], 56% [**ZVL**]) [PR]	GAP **OR: 0.07 [0.01-0.63] [ZV]** [PR]<br>GAP: 55% **[SIV, ZVL, PNV]**<br>Uncertainty: [**SIV** (75%)], [**PNV** (55%)], [**ZVL** (20%)]	Safe (majority) [PR]	-- -- --	-- -- --	-- -- --	-- -- --	-- -- --
**Yilmaz-Karadağ F et al. [27]**	-- -- --	-- -- --	-- -- --	-- -- --	Perceived Risk [PR]	-- -- --	-- -- --	No Importance [PR]
**Akan H et al. [28]**	-- -- --	-- -- --	Safe **1.55<OR<2.45 Median (OR): 2.08**	Benefits **5.18<OR<9.29 Median (OR): 8.73**	Perceived Risk **1.77<OR<7.49 Median (OR): 4.27** [OV]	Trust in institutions **OR: 2.52 (1.36-4.67)**	Natural Protection **OR:0.39(0.27-0.57)**	-- -- --
**Klett-Tammen CJ et al. [29]**	Official Sources (85%), Sufficient Information (90%),	Good Knowledge (97%)	-- -- --	-- -- --	-- -- --	-- -- --	-- -- --	-- -- --

Author	Data and data-sources	Knowledge	Safety	Efficacy/benefits	Perceived risk	Trust	Protection	Important
**Verger P et al. [30]**	Official Sources (80%)	-- -- --	Adverse drug reaction (6-11%) Safe (11%-86%)	No Efficacy (36%) [**HBVHPV, INF**]	-- -- --	No trust in institutions	-- -- --	-- -- --
**Flicoteaux R et al. [31]**	Official Sources **OR: 2.03[1.42-2.92]** Media News **OR: 0.57[0.41-0.79]**	-- -- --	Safe **OR:0.17 [0.09-0.31]** [PR] Adverse drug reaction (33%)	Efficacy **OR: 0.28[0.18-0.41]** [PR] No Efficacy (45%)	No Perceived Risk **OR:0.6[0.44-0.82]** No Perceived Risk (52%) [PR]	-- -- --	-- -- --	-- -- --
**Pulcini C et al. [32]**	Internet **OR: 0.92[0.85-0.99]** Official Sources (79%), Media News (32%) Continuing Education Courses **[INF]** Internet **[INF]**	-- -- --	-- -- --	-- -- --	-- -- --	-- -- --	-- -- --	-- -- --
**Pulcini C et al. [33]**	Official Sources **OR: 7.37[1.15-47.11]** Official Sources (97%), Internet (79%)	Misconception	-- -- --	No Efficacy (11%)	No Danegrous Disease **OR: 0.32[0.14-0.72] [MEAS]** [PR]	-- -- --	-- -- --	-- -- --
**Verger P et al. [34]**	Official Sources **p<0.0001** No Media News **p<0.0001** [OV]	-- -- --	Safe **p<0.0001** Adverse drug reaction (33%)	Efficacy **p<0.0001**	Perceied Risk, Dangerous Disease **p<0.0001**	Trust in institutions **P<0.0001** No Trust in data (45%) [OV]	-- -- --	Importance (67%)

NO *statistical hypothesis testing between* KBAB and *own vaccination and patient recommendation*

Author	Data and data-sources	Knowledge	Safety	Efficacy/benefits	Perceived risk	Trust	Protection	Important
**Deruelle et al. [35]**	Lack of information (12%-20%) [OV] Lack of information (12%-89%) [PR]	-- -- --	Adverse drug reaction (77%) [OV] Adverse drug reaction (17%) [PR]	No efficacy (28%) [OV]	-- -- --	-- -- --	-- -- --	-- -- --

Author	Data and data-sources	Knowledge	Safety	Efficacy/benefits	Perceived risk	Trust	Protection	Important
**Bayliss J et al. [36]**	-- -- --	-- -- --	-- -- --	-- -- --	Perceived Risk [PR]	-- -- --	-- -- --	-- -- --
**Hurley LP et al. [37]**	-- -- --	Good Knowledge (58%) GAP	-- -- --	-- -- --	-- -- --	-- -- --	-- -- --	-- -- --
**Napolitano F et al. [38]**	Scientific Journals (72%), SM (43%), C (19%), Internet (31%), [PR] Lack of information (40%) [PR]	-- -- --	Safe (68%) Adverse drug reaction (18%) [PR]	Efficacy (69%) No Efficacy ♂ (21%) [PR]	-- -- --	-- -- --	Protection (22%) [PR]	-- -- --
**Celep G et al. [39]**	Lack of information[PR]	-- -- --	Adverse drug reaction [PR]	-- -- --	Perceived Risk [PR]	-- -- --	-- -- --	Importance [PR]
**Kalemaki D et al. [40]**	-- -- --	-- -- --	Adverse drug reaction (8%) [OV]	No Efficacy (4%) [OV]	No Perceived Risk (27%) [OV]	-- -- --	Natural Protection (4%) [OV]	Importance [OV]
**Meites E et al. [41]**	Insufficient Information (30-37%) [PR]	Good Knowledge (58-63%) [PR]	-- -- --	-- -- --	-- -- --	-- -- --	-- -- --	-- -- --
**Awadlla NJ et al. [42]**	-- -- --	Misconception (16%) [**INF**]	-- -- --	-- -- --	-- -- --	-- -- --	-- -- --	-- -- --
**Collange F et al. [43]**	Official Sources, Personal Experiences, Insufficient Information [PR]	-- -- --	Adverse drug reaction [PR]	No Efficacy [PR]	-- -- --	-- -- --	-- -- --	-- -- --
**Glavier M et al. [44]**	Insufficient Information (35%) [PR]	-- -- --	-- -- --	No Efficacy (7%) [PR]	-- -- --	-- -- --	-- -- --	-- -- --
**Hurley LP et al. [45]**	-- -- --	GAP: (79-95%) **[RVZ, ZVL]** MI **[ZVL]**	Adverse drug reaction: [**ZVL]**	Efficacy [**ZVL]**	Perceived Risk **[ZVL]** **[PR]**	-- -- --	-- -- --	-- -- --

Author	Data and data-sources	Knowledge	Safety	Efficacy/benefits	Perceived risk	Trust	Protection	Important
			Adverse drug reaction: **[RZV]** [PR]					
**Le Marechal M et al. [46]**	Media News, Insufficient Information	-- -- --	Adverse drug reaction (16%-32%) Safe (52%-84%)	-- -- --	-- -- --	-- -- --	-- -- --	-- -- --
**Levi M et al. [47]**	-- -- --	GAP	Adverse drug reaction (33-67%)	-- -- --	-- -- --	-- -- --	-- -- --	Importance (48%-51%) No Importance: 1%
**Merriel SWD et al. [48]**	-- -- --	GAP (79%) [PR]	-- -- --	-- -- --	No Perceived Risk (50%) [PR]	-- -- --	-- -- --	-- -- --
**Steben M et al. [49]**	Official Sources (88%)	GK (majority) GAP (<10%)	Safe (majority) Adverse drug reaction (<10%)	Benefits (majority) No Benefits (<10%) [PR]	-- -- --	-- -- --	-- -- --	-- -- --
**Desiante F et al. [50]**	-- -- --	-- -- --	Adverse drug reaction (6%-35%) [OV]	-- -- --	No Perceived Risk (58%)	-- -- --	Self- Protection (>50%) [OV], Patient Protection (45%), Protection family and friend (35%)	-- -- --
**Hurley LP et al. [51]**	-- -- --	GK: Majority **[INF, PPSV23, ZV]** GAP [PR]	-- -- --	-- -- --	-- -- --	-- -- --	-- -- --	-- -- --
**Hurley LP et al. [52]**	Official Sources [PR]	GAP (25-29%) + MI	-- -- --	-- -- --	-- -- --	-- -- --	-- -- --	Importance **[INF, PNV, Tdap/Td]** / NO IM **[INF, Tdap/Td, ZV]**
**Raude J et al. [53]**	Official Sources (75%)	-- -- --	Safe Adverse drug reaction Adverse Adverse drug	Benefits (majority) No Benefits (minority)	Perceived Risk (50%) [PR]	No trust in industry (50%)	-- -- --	-- -- --

Author	Data and data-sources	Knowledge	Safety	Efficacy/benefits	Perceived risk	Trust	Protection	Important
			reaction (Minority)					
**Verger P et al. [54]**	Personal Experiences, Internet, Media News > Continuing Education Courses	GAP	Safe (11%-86%) Adverse drug reaction (3%-11%)	Efficacy+ Benefits (11%-86%), No Efficacy/ Benefits (3%-11%)	-- -- --	-- -- --	-- -- --	-- -- --
**Massin S et al. [55]**	-- -- --	-- -- --	-- -- --	Benefit - Risk	Perceived Risk [OV]+[PR]	-- -- --	-- -- --	-- -- --
**Alsaleem MA [56]**	Co-workers (58%) [OV]	GAP	Adverse drug reaction (50%) [OV]	No Efficacy	Perceived Risk (61%) [OV] No Perceived Risk (25%) [OV]	No Trust (47%-64%)	Self-Protection (74%), Patient Protection (61%), Protection Family and friend (77%) [OV]	No Importance
**François M et al. [57]**	-- -- --	GAP, Good Knowledge	Safe (80%) Adverse drug reaction (65%) [PR]	-- -- --	No Perceived Risk (25%) [PR]	No Trust in data	Previous protection	-- -- --
**Inoue Y et al. [58]**	-- -- --	-- -- --	-- -- --	-- -- --	-- -- --	-- -- --	-- -- --	-- -- --
**Lutringer-Magnin D et al. [59]**	Scientific Journals (68%), Pharmaceutical Representatives (70%), Official Sources (9%)	Misinterpretation (29%) [PR]	-- -- --	Efficacy (15%) Benefits (60%) [PR]	-- -- --	No Ttust in data (43%) [PR]	-- -- --	-- -- --
**Rurik I et al. [60]**	Media News > Official Sources	-- -- --	Safe (40%) Adverse drug reaction (59%)	Efficacy (67%-86%)	No Perceived Risk [PR]	Trust in institutions < Trust in media	-- -- --	-- -- --
**Ward K et al. [61]**	-- -- --	Good Knowledge (73%) GAP (10%)	Safe(90%) Adverse drug reaction (7%) [OV]	Efficacy (90%) No Benefits (10%)	Perceived Risk[OV] No Perceived Risk (10%)	-- -- --	Patient Protection (94%)	-- -- --

Author	Data and data-sources	Knowledge	Safety	Efficacy/benefits	Perceived risk	Trust	Protection	Important
		Misconception (1%)						

***Table S1**: Intrinsic and extrinsic factors reported by each study and their influence on vaccination/recommendation.*

***[OV]**: Own vaccination; **[PR]**: patient recommendation; **INF** (influenza); **HPV** (human papillomavirus vaccine); **HBV** (hepatitis B); **MEAS** (measles); **MMR** (measles, mumps and rubella); **PER** (pertussis); **PNV** (pneumococcal vaccine); **PPSV23** (pneumococcal polysaccharide vaccine); **ZV** (zoster vaccine); **RVZ** (recombinant zoster vaccine); **ZVL** (zoster live-attenuated vaccine); **TT** (tetanus vaccine); **Td** (tetanus-diphtheria); **Tdap** (tetanus, diphtheria, pertussis).*

Author	Responsability	Attitude	Extrinsic factors	Practices (behaviours)	Experiences
			Statistical hypothesis testing between KBAB and own vaccination and patient recommendation		
**Verger P et al. [21]**		-- -- --	-- -- --	-- -- --	-- -- --
**Verger P et al. [22]**	-- -- --	-- -- --	-- -- --	-- -- --	-- -- --
**Arlt J et al. [23]**	-- -- --	Attitude +	Organisational Factor **OR: 4.31[1.12-16.60]** [P]<br>Patient Factor **OR: 3.20[1.89-5.43]**<br>Organisational Factor, Financial Barrier: (3%-31%)	Check vaccination satatus	-- -- --
**Neufeind J et al. [24]**	Responsability	-- -- --	Organizational Barrier: (25.4%-41%) **[MEAS]** [R]<br>Organizational Barrier: **[INF, PER, HBV]** [P]	Vaccination History **OR: 44.09[8.93-799.89] [INF]** [R]<br>Discuss Vaccination (59.6%-87.4%), CVS (81.2%), RS (40%)	-- -- --
**Verhees RAF et al. [25]**	Responsability **OR: 1.82 [1.32-2.64] [INF, PER, HBV]** [OV]	-- -- --	-- -- --	-- -- --	-- -- --

Author	Responsability	Attitude	Extrinsic factors	Practices (behaviours)	Experiences
**Vezzosi L et al. [26]**	-- -- --	Attitude + **OR: 13.67 [2.41-77.64]** [PNV, ZV] [R]<br>Attitude +: [**SIV, PNV, ZV]**	-- -- --	Vaccination history **OR: 5.44 [1.08-27.31] [PNV]** [R]<br>Vaccination History **OR: 19.36[2.60-139.61] [ZV]** [R]	Patient Experience **OR: 6.61[1.11-44.43] [ZV]** [R]
**Yilmaz-Karadağ F et al. [27]**	-- -- --	-- -- --	-- -- --	Vaccination History **p>0.05 [HBV, INF, Td]** [R]	-- -- --
**Akan H et al. [28]**	-- -- --	Attitude + **3.06<OR<10.9 Median (OR): 7.245**	Organisational factor **2.64<OR<13.75 Median (OR): 6.57**	Vaccination History **OR: 15.14[2.06-11.25]**<br>Reminder System **OR:1.66[1.15-2.38]**	-- -- --
**Klett-Tammen CJ et al. [29]**	-- -- --	-- -- --	-- -- --	-- -- --	-- -- --
**Verger P et al. [30]**	No Responsability (7%)	-- -- --	-- -- --	Vacciantion History **OR: 2.95[2.31-3.77] [INF]** [R]<br>Vaccination History **OR: 1.90[1.27-2.84] [HBV]** [R]<br>Discuss Vaccination (43%) [R]	-- -- --
**Flicoteaux R et al. [31]**	-- -- --	-- -- --	-- -- --	-- -- --	Patient Experience **OR: 2.81[1.98-3.99]** [R]<br>Patient Experience (30.2%)
**Pulcini C et al. [32]**	-- -- --	-- -- --	-- -- --	Vaccination History 1**.08<OR<3.08 Mediana (OR): 1.2** [P]<br>Discuss Vaccination	-- -- --
**Pulcini C et al. [33]**	-- -- --	Attitude + (96%)	Patient Barrier: **(46%) [R]**<br>Organisational Barrier: (16-48%) [R]	Check Vaccination Status **OR: 3.38[1.50-7.62]**<br>Discuss Vaccination (80%)	-- -- --
**Verger P et al. [34]**	-- -- --	-- -- --	Organisational Barrier: 7% [P]	Vaccination History (14.5%) [P]	-- -- --
NO ***statistical hypothesis testing between*** **KBAB and** ***own vaccination and patient recommendation***					
**Deruelle et al. [35]**	-- -- --	-- -- --	-- -- --	-- -- --	-- -- --

Author	Responsability	Attitude	Extrinsic factors	Practices (behaviours)	Experiences
**Bayliss J et al. [36]**	-- -- --	-- -- --	-- -- --	Discuss Vaccination (9-85%)	-- -- --
**Hurley LP et al. [37]**	-- -- --	-- -- --	-- -- --	Store vaccines (74%)	-- -- --
**Napolitano F et al. [38]**	-- -- --	-- -- --	-- -- --	Collect Informatino Practices (32%♀, 28%♂) [R]<br>Check vaccination satuts (2.7%-13.4% ♂)	-- -- --
**Celep G et al. [39]**	-- -- --	-- -- --	-- -- --	Discuss Vaccination [**INF** (94%)], [**HBV** (37%)], [**Tdap** (14%)].	No Own Experience [R]
**Kalemaki D et al. [40]**	-- -- --	-- -- --	Organisational factor: (47%) [P]	-- -- --	Own Experience [P]
**Meites E et al. [41]**	-- -- --	-- -- --	-- -- --	-- -- --	-- -- --
**Awadlla NJ et al. [42]**	Responsability (61%) [OV]	-- -- --	-- -- --	-- -- --	-- -- --
**Collange F et al. [43]**	Responsability [PR]	Attitude + [R]	-- -- --	-- -- --	Patient Controversies +<br>Own Experience [R]
**Glavier M et al. [44]**	-- -- --	Attitude +<br>(92.6%) [R]	Organisational Factor OB: (3.3%-14.4%) [R]	-- -- --	-- -- --
**Hurley LP et al. [45]**	-- -- --	Attitude + **[ZVL]**	Financialf acilitator (90%) **ZVL** [R]<br>(40%) **RZV** [R]<br>Patient Factor: (19%) **ZVL** [R]<br>Organisational Barrier: (35%) **ZVL** [R]	-- -- --	-- -- --
**Le Marechal M et al. [46]**	-- -- --	-- -- --	-- -- --	-- -- --	-- -- --
**Levi M et al. [47]**	-- -- --	Attitude +	Organisational Barrier: (70.7%) [R]	-- -- --	-- -- --
**Merriel SWD et al. [48]**	-- -- --	-- -- --	Organisational Barrier: (65.70%) [R]	Collect Information Pracitces (86.96%) [R]	-- -- --
**Steben M et al. [49]**	-- -- --	-- -- --	-- -- --	Discuss Vaccination [HPV]	-- -- --

Author	Responsability	Attitude	Extrinsic factors	Practices (behaviours)	Experiences
Desiante F et al. [50]	-- -- --	-- -- --	-- -- --	-- -- --	-- -- --
Hurley LP et al. [51]	-- -- --	-- -- --	Financial Facilitator: [R]	-- -- --	-- -- --
Hurley LP et al. [52]	-- -- --	Attitude +	-- -- --	Discuss Vaccination	-- -- --
Raude J et al. [53]	-- -- --	-- -- --	-- -- --	Disuss Vaccination	-- -- --
Verger P et al. [54]	-- -- --	Attitude + (17%-80%)	-- -- --	Disuss Vaccination (90%)	Own Experience
Massin S et al. [55]	-- -- --	Attitude + (69%)	-- -- --	-- -- --	-- -- --
Alsaleem MA [56]	-- -- --	-- -- --	Organisational Barrier: (25%) [P]	-- -- --	-- -- --
François M et al. [57]	-- -- --	-- -- --	Organisational Barrier: (5-13%)<br>Patient Facilitator: (11%)	Disuss Vaccination (12%)	-- -- --
Inoue Y et al. [58]	-- -- --	-- -- --	Financial Facilitator: 32% [R]	Vaccination History [R]	Past Controversies + Own Experience[R]
Lutringer-Magnin D et al. [59]	-- -- --	Attitude + (17.4%-80.8%)<br>Attitude - (1.8%-17.4%)	-- -- --	Discuss Vaccination 19-23 years (52.6%♀) [R]	-- -- --
Rurik I et al. [60]	-- -- --	-- -- --	Organisational Barrier: 69%	-- -- --	-- -- --
Ward K et al. [61]	-- -- --	-- -- --	Financial Factor: (25%) [P]	-- -- --	-- -- --
