# Supplementary material for: Understanding Primary Care Physician Vaccination Behaviour: A Systematic Review
Source: Int J Environ Res Public Health. 2022 Oct 25;19(21):13872. doi: 10.3390/ijerph192113872 (PMC9654811; doi:10.3390/ijerph192113872)
Supplement: Supplementary file 1 [file ijerph-19-13872-s001.zip › Supplementary Material S2.pdf]

**Table S2:** Quality NOS * assessment, * Newcastle-Ottawa scale for cross-sectional studies.

| | Selection | | | | | | | | | | | | Comparability | | Outcome | | | | | |
|---|---|---|---|---|---|---|---|---|---|---|---|---|---|---|---|---|---|---|---|---|
| | 1 | | | | 2 | | 3 | | | 4 | | | 1 | | 1 | | | 2 | | |
| | a) | b) | c) | d) | a) | b) | a) | b) | c) | a) | b) | c) | a) | b) | a) | b) | c) | a) | b) | TOTAL SCORE |
| **Verger P et al. [21]** | * | | | | | * | | | * | | | | * | * | | | * | * | | **7** |
| **Verger P et al. [22]** | * | | | | | * | | | * | | | | * | * | | | * | * | | **7** |
| **Arlt J et al. [23]** | * | | | | | | * | | | ** | | | | * | | | * | * | | **7** |
| **Neufeind J et al. [24]** | * | | | | | | | | | ** | | | | * | | | * | * | | **6** |
| **Verhees RAF et al. [25]** | * | | | | | * | | | | ** | | | | * | | | | | | **5** |
| **Vezzosi L et al. [26]** | * | | | | | | | | | * | | | | | | | * | | | **3** |
| **Yilmaz-Karadağ F et al. [27]** | * | | | | | | | | | ** | | | | * | | | * | | | **5** |
| **Akan H et al. [28]** | | | | | | | | | | ** | | | | | | | * | * | | **4** |
| **Klett-Tammen CJ et al. [29]** | * | | | | | | | | | ** | | | * | | | | * | * | | **6** |
| **Verger P et al. [30]** | * | | | | * | | * | | | ** | | | | | | | * | | | **6** |
| **Flicoteaux R et al. [31]** | * | | | | | | | | | * | | | | | | | * | * | | **4** |
| **Pulcini C et al. [32]** | * | | | | | | | | | ** | | | | | | | * | * | | **5** |
| **Pulcini C et al. [33]** | * | | | | | | | | | ** | | | | | | | | | | **3** |
| **Verger P et al. [34]** | * | | | | | | * | | | ** | | | | | | | * | * | | **6** |
| **Deruelle et al. [35]** | * | | | | | | | | | * | | | | * | | | * | * | | **5** |
| **Bayliss J et al. [36]** | * | | | | | | | | | | | | | * | | | * | | | **3** |
| **Hurley LP et al. [37]** | * | | | | | | * | | | * | | | | | | | * | | | **4** |
| **Napolitano F et al. [38]** | * | | | | * | | | | | ** | | | | * | | | * | * | | **7** |
| **Celep G et al. [39]** | | | | | | | | | | ** | | | | | | | * | | | **3** |
| **Kalemaki D et al. [40]** | * | | | | | | | | | * | | | | | | | * | * | | **4** |
| **Meites E et al. [41]** | * | | | | | | * | | | ** | | | | | | | * | | | **6** |
| **Awadlla NJ et al. [42]** | * | | | | | | | | | ** | | | | * | | | * | * | | **6** |
| **Collange F et al. [43]** | * | | | | | | * | | | ** | | | | | | | * | | | **5** |

| | Selection | | | | | | | | | | | | Comparability | | Outcome | | | | | |
|---|---|---|---|---|---|---|---|---|---|---|---|---|---|---|---|---|---|---|---|---|
| | **1** | | | | **2** | | **3** | | | **4** | | | **1** | | **1** | | | **2** | | |
| | **a)** | **b)** | **c)** | **d)** | **a)** | **b)** | **a)** | **b)** | **c)** | **a)** | **b)** | **c)** | **a)** | **b)** | **a)** | **b)** | **c)** | **a)** | **b)** | **TOTAL SCORE** |
| **Glavier M et al. [44]** | * | | | | * | | | | | * | | | | | | | * | * | | **5** |
| **Hurley LP et al. [45]** | * | | | | | | * | | | ** | | | | | | | * | | | **5** |
| **Le Marechal M et al. [46]** | * | | | | | | | | | ** | | | | | | | * | * | | **5** |
| **Levi M et al. [47]** | * | | | | | | | | | | * | | | | | | * | * | | **4** |
| **Merriel SWD et al. [48]** | | | | | | | | | | | | | | | | * | | * | | **5** |
| **Steben M et al. [49]** | | | | | * | | | | | | | | | | | * | | | | **5** |
| **Desiante F et al. [50]** | | | | | | | | | | | | | | | | * | | * | | **3** |
| **Hurley LP et al. [51]** | | | | | | | | | | | | | | | | * | | | | **4** |
| **Hurley LP et al. [52]** | | | | | | | | | | | | | | | | * | | * | | **7** |
| **Raude J et al. [53]** | | | | | | | | | | | | | | | | * | | * | | **7** |
| **Verger P et al. [54]** | | | | | | | | | | | | | | | | * | | * | | **6** |
| **Massin S et al. [55]** | | | | | | | | | | * | | | | | | * | | | | **3** |
| **Alsaleem MA [56]** | | | | | | | | | | | | | | | | * | | | | **4** |
| **François M et al. [57]** | * | | | | | | | | | | | | | | | * | | | | **4** |
| **Inoue Y et al. [58]** | | | | | | | | | | | | | * | | | * | | | | **6** |
| **Lutringer-Magnin D et al. [59]** | | | | | | | | | | | | | | | | * | | * | | **6** |
| **Rurik I et al. [60]** | | | | | | | | | | | | | * | | | * | | | | **3** |
| **Ward K et al. [61]** | | | | | | | | | | | | | | | | * | | | | **4** |

***Selection***. 1)Representativeness of the sample; 2) Sample size; 3) Non-respondents; 4) Ascertainment of exposure (risk factor). ***Comparability***. 1) Subjects in different outcome groups are comparable, based on the study design or analysis. Confounding factors are controlled for.

***Outcome.*** 1) Assessment of the outcome; 2) Statistical test.
