# Supplementary material for: Understanding Primary Care Physician Vaccination Behaviour: A Systematic Review
Source: Int J Environ Res Public Health. 2022 Oct 25;19(21):13872. doi: 10.3390/ijerph192113872 (PMC9654811; doi:10.3390/ijerph192113872)
Supplement: Supplementary file 1 [file ijerph-19-13872-s001.zip › Supplementary Material S3.pdf]

Search strategy:

(attitude* OR knowle* OR percept* OR behaviour* OR conduct* OR understand* OR belief* OR barrier* OR hesitancy*) AND (vaccin* OR inoculat*) AND (primary healthcare physician* OR general practitioner* OR family physician*) AND (“cross-sectional OR” OR “survey” OR “cohort OR” OR “case-control” OR “OR” OR “adjusted OR”).
